# Identifying core competencies for community oral health practice among dental hygienists: a scoping review

**DOI:** 10.3389/fpubh.2026.1877748

**Published:** 2026-09-09

**Authors:** Su-Kyung Park, Jung Yun Kang, Sun-Jung Shin, Sun-Mi Lee, Nam-Hee Kim

**Affiliations:** 1 Department of Dental Hygiene, Graduate School, Yonsei University Mirae Campus, Wonju, Republic of Korea; 2 Department of Dental Hygiene, Yonsei University Mirae Campus, Wonju, Republic of Korea; 3 Department of Dental Hygiene, College of Dentistry, Kangwon National University, Gangneung, Republic of Korea; 4 Department of Dental Hygiene, Dongnam Health University, Suwon, Republic of Korea

**Keywords:** competency-based education, dental hygienists, health promotion, oral health, public health

## Abstract

**Background:**

Community oral health practice increasingly requires dental hygienists to perform prevention-oriented, population-based, and interprofessional roles beyond traditional chairside clinical care. However, existing competency frameworks primarily emphasize clinical practice or general professional competencies, and an integrated competency framework specifically designed for community oral health practice remains unavailable. This study aimed to identify core competencies for community oral health practice among dental hygienists through a scoping review.

**Methods:**

A scoping review was conducted in accordance with the JBI methodology and reported following the PRISMA-ScR guidelines. Literature published between 2005 and 2025 was identified through PubMed, Scopus, Google Scholar, and targeted searches of official organizational websites. Following predefined eligibility criteria, 38 sources were synthesized across three analytical layers: (1) seven cross-professional competency frameworks represented by 12 source documents, (2) five national or regional dental hygiene competency frameworks represented by nine source documents, and (3) 17 community oral health practice literature sources. Competency domains were identified through iterative qualitative coding and retained when supported by convergent evidence across at least two of the three analytical layers.

**Results:**

Eight integrated competency domains were identified: Health Equity Practice (based on social determinants of health, SDOH); Population-Based Program Planning, Implementation & Evaluation; Professionalism & Social Responsibility; Policy & Health System Literacy; Community Problem-Solving; Interprofessional Collaboration & Leadership; Evidence-Based Practice & Data Literacy; and Cultural Competence & Public Communication. The resulting framework integrates competencies derived from international healthcare professional frameworks, national dental hygiene competency frameworks, and community oral health practice literature, reflecting the expanding public health role of dental hygienists.

**Conclusion:**

This study proposes an integrated competency framework for community oral health practice that may support competency-based curriculum development, workforce preparation, and professional development for dental hygienists. The framework provides a structured foundation for strengthening prevention-focused community oral health practice and may be adapted and validated across diverse educational, sociocultural, and healthcare settings.

## 1 Introduction

Global health is challenged by population aging, the rapid rise in chronic diseases, and persistent health inequities. Oral diseases remain among the most prevalent conditions worldwide and disproportionately burden socially disadvantaged populations despite being largely preventable. This discrepancy between preventability and actual outcomes highlights the limitations of treatment-centered care and underscores the need for workforce capacity capable of delivering prevention and population-level interventions in community settings. Consequently, oral health professionals are expected to contribute not only to patient treatment in clinical settings, but also to health promotion, risk reduction, and responses to social determinants of health within community settings (1, 2).

As one of the principal oral health professional groups, dental hygienists are recognized as primary care providers whose scope of practice bridges clinical prevention and community-based oral health promotion, enabling them to support prevention and health promotion across the lifespan and contribute to improving oral health at both individual and community levels (3). However, education and training systems in many countries remain predominantly procedure-focused, resulting in a mismatch between workforce preparation and public health demands (4). Dental hygienists increasingly participate in a broad range of prevention-oriented and community-based activities, including program planning, community outreach, and services for vulnerable populations. However, these activities are often undertaken without clearly defined competency standards (5). Existing competency models emphasize clinical proficiency or general professional abilities rather than competencies related to prevention-focused community oral health practice, and competencies such as interprofessional collaboration, cultural responsiveness, digital health literacy, and policy engagement are not consistently integrated into education (6, 7).

Previous studies have attempted to define competencies relevant to dental hygiene and public health practice. Dental education research has emphasized the need to move beyond procedure-based training toward prevention and community engagement, while public health competency frameworks have identified leadership, communication, collaboration, and professionalism as essential workforce capacities (4, 6). In dental hygiene, community-based education and project-based learning have been shown to improve perceived competence among students (8). However, these studies primarily address educational outcomes or general professional competencies and do not provide an integrated competency framework specifically designed for community oral health practice (9). Furthermore, many existing frameworks have been developed within single national contexts, limiting their applicability to diverse healthcare systems and restricting cross-national workforce development (10, 11).

Building on these developments, dental hygiene practice has expanded internationally in response to increasing demands for prevention-focused community oral health services (12). Despite this expansion, competency frameworks have evolved unevenly across countries because of differences in healthcare systems, educational structures, and professional regulation (10, 11). Consequently, competency expectations remain inconsistently defined, limiting workforce comparability and the transferability of educational models (13, 14). Establishing internationally relevant competencies is therefore necessary to support comparable workforce preparation and facilitate the implementation of community oral health practice across diverse healthcare and public health settings (15, 16).

Given the heterogeneous and fragmented nature of existing competency evidence across diverse professional and national frameworks—and the absence of an established and integrated competency framework for community oral health practice—a scoping review was selected as the most appropriate methodology for this study (17). Scoping reviews are particularly well suited to mapping broad bodies of literature where key concepts remain inconsistently defined and where the goal is to identify and synthesize evidence from diverse sources rather than to evaluate specific interventions (18). The review was conducted in accordance with the Joanna Briggs Institute (JBI) methodology for scoping reviews and reported following the Preferred Reporting Items for Systematic Reviews and Meta-Analyses Extension for Scoping Reviews (PRISMA-ScR) guidelines to ensure methodological rigor and transparency (17, 20).

Therefore, this study aimed to identify core competencies for community oral health practice among dental hygienists through a scoping review synthesizing international healthcare professional competency frameworks, national dental hygiene competency frameworks, and community oral health practice literature. The findings of this study may serve as foundational evidence for competency-based education policies and workforce development strategies in educational and professional settings similar to those represented in the included sources. The proposed framework may inform context-specific adaptation for strengthening population-level oral health promotion, prevention-focused community oral health practice, and the expanding public health role of dental hygienists.

## 2 Materials and methods

### 2.1 Study design

This study was conducted as a scoping review to identify competency domains required for community oral health practice among dental hygienists. The review followed the methodological framework proposed by Arksey and O’Malley (19), further refined by Peters et al. (20), and was reported in accordance with the Preferred Reporting Items for Systematic Reviews and Meta-Analyses Extension for Scoping Reviews (PRISMA-ScR) guidelines.

A scoping review was selected as the most appropriate methodology for this study for several reasons. First, scoping reviews are specifically designed to map the extent, range, and nature of evidence on a topic, identify key concepts and definitional gaps, and synthesize findings across heterogeneous sources where no dominant framework has been established (19, 20). As competency frameworks related to community oral health practice among dental hygienists have developed unevenly across countries and professional contexts, a scoping review was considered more appropriate for this exploratory objective than a systematic review.

Second, unlike a systematic review—which evaluates the effectiveness of specific interventions through critical appraisal and effect synthesis—the present study aimed to identify and map competency domains across diverse frameworks rather than assess intervention outcomes. Quality appraisal procedures used in systematic reviews are primarily designed for intervention research and are less suitable for synthesizing heterogeneous competency frameworks, policy documents, and educational guidelines.

Third, unlike a narrative review, a scoping review follows a pre-specified, transparent, and reproducible methodology, including comprehensive database searches, independent dual-reviewer screening, and structured data charting, thereby minimizing selection bias and enhancing methodological rigor. Accordingly, the scoping review methodology provided both the flexibility and systematic rigor required to derive an integrated competency framework that may inform context-specific adaptation in educational and professional settings similar to those represented in the included sources.

A review protocol was not formally registered prior to the study because the primary objective was exploratory evidence mapping rather than intervention-effect evaluation. However, to ensure methodological transparency and reproducibility, the eligibility criteria, screening procedures, and data-charting processes were predefined and documented prior to study selection, consistent with recommended best practices for conducting scoping reviews.

The review process followed five stages as outlined in the Arksey and O’Malley framework and updated JBI guidance (19, 20).

1. Identifying research questions,
2. Identifying relevant studies,
3. Study selection,
4. Data charting, and
5. Collating and summarizing the results.

### 2.2 Search strategy and eligibility criteria

#### 2.2.1 Research questions

The review addressed the following questions:

1. What competency domains are commonly reported across healthcare professional competency frameworks?
2. What competencies are described in dental hygiene competency frameworks across countries?
3. Which competencies are related to community-based and public oral health practice?
4. How can these competencies be structured into an integrated framework for community oral health practice?

In this review, *community oral health practice* refers to population-based prevention, health promotion, and community engagement activities delivered beyond individual chairside clinical care, including community programs, outreach services, interprofessional collaboration, and public health coordination. Within this context, dental hygienists are expected to perform expanded roles that extend beyond traditional clinical functions and require competencies related to prevention-focused practice, population health, communication, collaboration, and health system responsiveness.

#### 2.2.2 Information sources and search strategy

A comprehensive literature search was conducted between March 1 and April 10, 2025. The following electronic databases were searched: PubMed, Scopus, and Google Scholar. Google Scholar was used as a supplementary search source to identify potentially relevant publications and competency framework–related documents that may not have been indexed in the primary bibliographic databases. Search results were sorted by relevance, and the first 100 records were manually screened according to the predefined eligibility criteria. The Google Scholar search was conducted on April 10, 2025.

Search terms were combined using Boolean operators and included: “core competencies,” “professional competencies,” “health professions education,” “public health competency,” “dental hygiene,” “community oral health,” “interprofessional collaboration,” “advocacy,” “health equity,” “policy,” “cultural competence,” and “digital health.”

Reference lists of included sources were manually screened to identify additional relevant competency frameworks and policy documents. Authoritative textbooks identified during the review process were not included as evidence sources for competency synthesis but were cited selectively in the Discussion to provide contextual background where appropriate. In addition, targeted searches of official websites of professional associations and accreditation organizations were conducted to identify competency frameworks, guidelines, and policy documents not indexed in bibliographic databases (Supplementary Table S1). Complete database-specific search strategies, including database-specific search syntax, Boolean operators, phrase searching, field restrictions, and Google Scholar screening procedures, are provided in Supplementary Appendix S1.

#### 2.2.3 Eligibility criteria

Sources were included if they:

- Presented competency frameworks or competency-related education for healthcare professionals.
- Described competencies for dental hygienists or comparable oral health workforce.
- Addressed public, community, or population-based practice.
- Were journal articles, guidelines, or policy reports.
- Were published between 2005 and 2025.
- Were written in English or Korean.

Sources were excluded if they:

- Focused exclusively on clinical procedural skills.
- Lacked explicit competency descriptions.
- Were conference abstracts, books, or theses.
- Did not provide accessible full text.

For Layer 2 dental hygiene competency frameworks, five national or regional frameworks were purposively selected for comparative analysis: Korea (Korean Dental Hygienists Association; KDHA), the United States (American Dental Education Association; ADEA), Canada (Federation of Dental Hygiene Regulators of Canada; FDHRC), Australia (Australian Dental Council; ADC), and Europe (Common European Curriculum for Dental Hygiene; CECDH). Following the identification of candidate frameworks, selection was based on the following four predefined criteria: (1) the existence of a formally published and publicly accessible standalone competency framework specifically developed for dental hygienists, structured around competency domains with behavioral indicators; (2) geographic diversity to ensure representation across Asian, North American, and European healthcare systems and regulatory contexts; (3) language accessibility in English or Korean, consistent with the overall search criteria; and (4) explicit inclusion of community health, health promotion, or public health dimensions as identifiable competency components within the framework.

Although competency-related documents from Japan and the United Kingdom were identified and reviewed, they did not meet the predefined eligibility criteria for comparative competency-framework analysis. In Japan, available documents primarily emphasized educational guidance rather than standalone competency frameworks with explicit competency domains and behavioral indicators. In the United Kingdom, the GDC *Safe Practitioner Framework* (2024) did not present community oral health competencies as discrete analytical domains comparable to those included in this review. Consequently, these sources were excluded, which may have introduced selection bias and limited the geographic representativeness of the synthesis. Specifically, the exclusion of Japanese competency-related documents may have underrepresented regulatory and educational perspectives from East Asian contexts. This limitation is discussed further in Section 4.

In this study, the term *oral health professionals* is used broadly to refer to trained practitioners involved in oral health promotion, disease prevention, and oral healthcare delivery, including dentists, dental hygienists, dental therapists, and other allied oral health personnel. *Dental hygienists* specifically refer to licensed oral health professionals whose scope of practice includes both chairside preventive care and community oral health practice activities. Although oral health professionals collectively contribute to population oral health outcomes, this study focuses specifically on the competency requirements of dental hygienists within community oral health practice, reflecting their unique role at the intersection of clinical prevention, health promotion, and public health service delivery.

#### 2.2.4 Study selection

**Figure 1:**
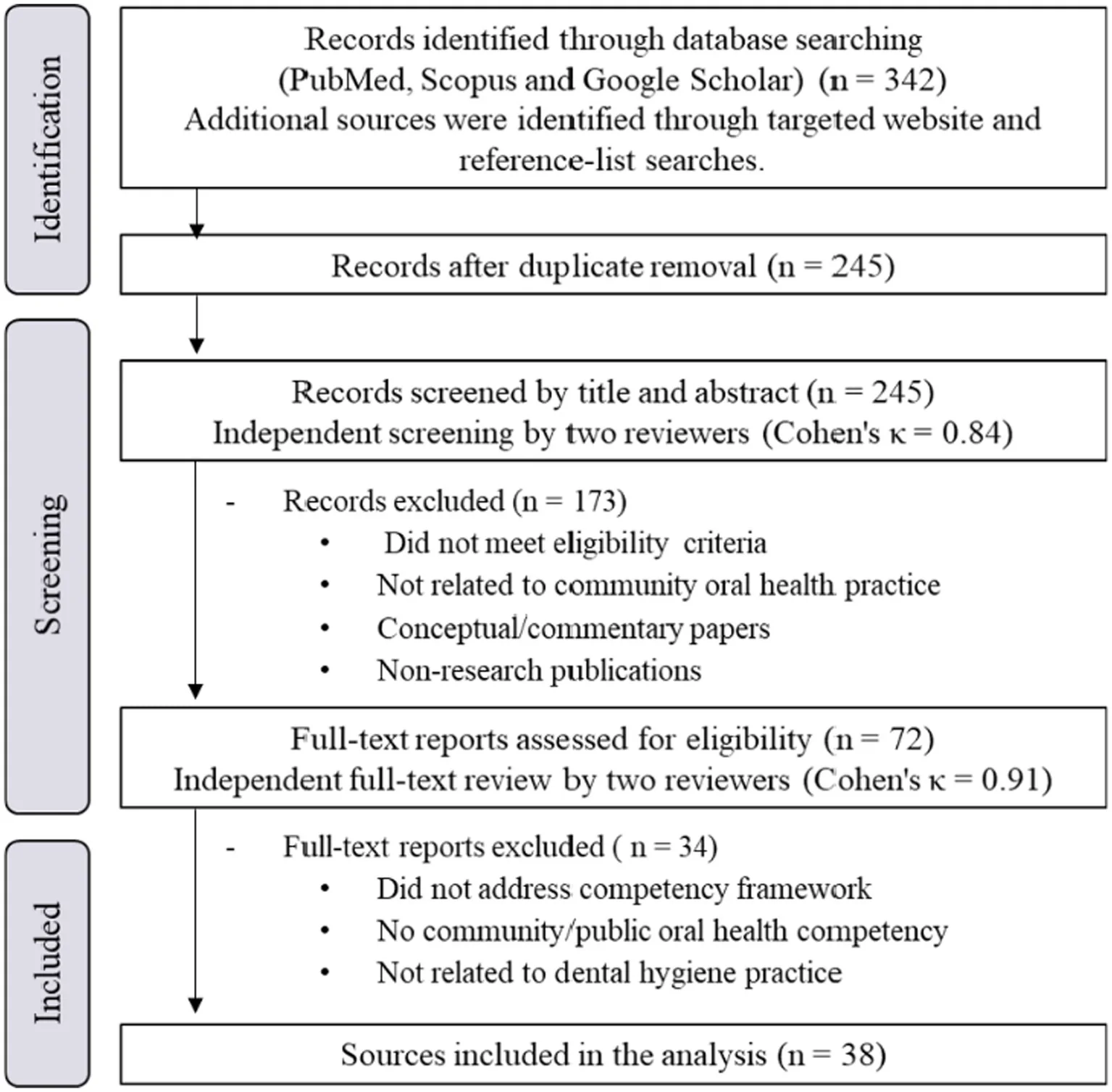
PRISMA-ScR flow diagram of the study selection process.

After removing 97 duplicates, 245 records were independently screened by two reviewers at the title and abstract level, and inter-rater agreement for this stage was substantial (Cohen’s *κ* = 0.84). Seventy-two full-text reports were then independently assessed for eligibility by two reviewers, and inter-rater agreement for this stage was almost perfect (Cohen’s *κ* = 0.91). Any discrepancies at either stage were resolved through discussion and consensus. Thirty-eight sources were included in the final synthesis. The selection process is presented in the PRISMA-ScR flow diagram (Figure 1) (46).

#### 2.2.5 Selection of national competency frameworks

Candidate national and regional dental hygiene competency frameworks were first identified through targeted searches of professional association, accreditation organization, and regulatory body websites, together with screening of reference lists in included sources. The identified candidate frameworks were subsequently assessed against the four predefined eligibility criteria described in Section 2.2.3 to determine their inclusion in Layer 2 of the synthesis.

#### 2.2.6 Data charting and coding process

A standardized data charting form was developed prior to analysis to extract competency-related information from each included document. Extracted items included publication characteristics, competency domain labels, definitions, behavioral indicators, and contextual descriptions.

Two reviewers independently performed pilot coding on five source documents to establish a shared coding framework. The coding scheme was iteratively refined through comparison and discussion. After calibration, all included sources were independently charted and cross-checked to ensure consistency. Competency-domain categorization was conducted independently by the two reviewers, and any discrepancies were resolved through iterative discussion and consensus until agreement was achieved. Because competency-domain categorization involved interpretive qualitative synthesis and iterative conceptual refinement rather than discrete binary screening decisions, Cohen’s kappa was not considered methodologically appropriate for this stage.

An initial open coding process identified recurring competency concepts across documents. Subsequently, axial coding grouped conceptually similar competencies into higher-order domains.

When multiple source documents were available for a single national competency framework, competency concepts were extracted from each document and subsequently synthesized at the framework level. Conceptually overlapping competency statements within the same framework were merged and counted once to minimize duplication while preserving the breadth of competency concepts.

The resulting categories were mapped across three analytical layers:

1. Healthcare professional competency frameworks,
2. National dental hygiene competency frameworks, and
3. Public oral health practice literature.

Competency domains were retained when supported by convergent evidence across at least two of the three analytical layers. Evidence mapping for each domain across the three analytical layers is provided in Supplementary Tables S2–S4.

#### 2.2.7 Data synthesis

For clarity, framework refers to a formally published competency document with a defined domain structure, whereas source refers to an individual publication included in the analysis. Because multiple publications may describe a single framework, the number of sources may exceed the number of frameworks reviewed.

Data synthesis consisted of three steps:

1. Descriptive analysis of study characteristics and competency structures
2. Thematic categorization of competency domains
3. Cross-framework comparative integration

Derived competencies were synthesized into an integrated competency framework for community oral health practice among dental hygienists.

Evidence locations were recorded at the domain or section level rather than page-level citation to ensure traceability while maintaining feasibility within a scoping review approach.

Competency domains were retained for inclusion in the integrated framework if they were supported by evidence from at least two of the three analytical layers (≥ 2 of 3). This threshold was selected for two reasons: first, to prevent inclusion of competency domains supported by only a single layer of evidence, which could reflect idiosyncratic disciplinary or contextual priorities rather than convergent competency expectations across multiple evidence sources; second, to avoid the overly restrictive criterion of requiring support across all three layers simultaneously, which would risk excluding competencies that are genuinely relevant but structurally underrepresented in one layer due to differences in document type and scope across the three layers.

## 3 Results

### 3.1 Study selection and overview

**Figure 2:**
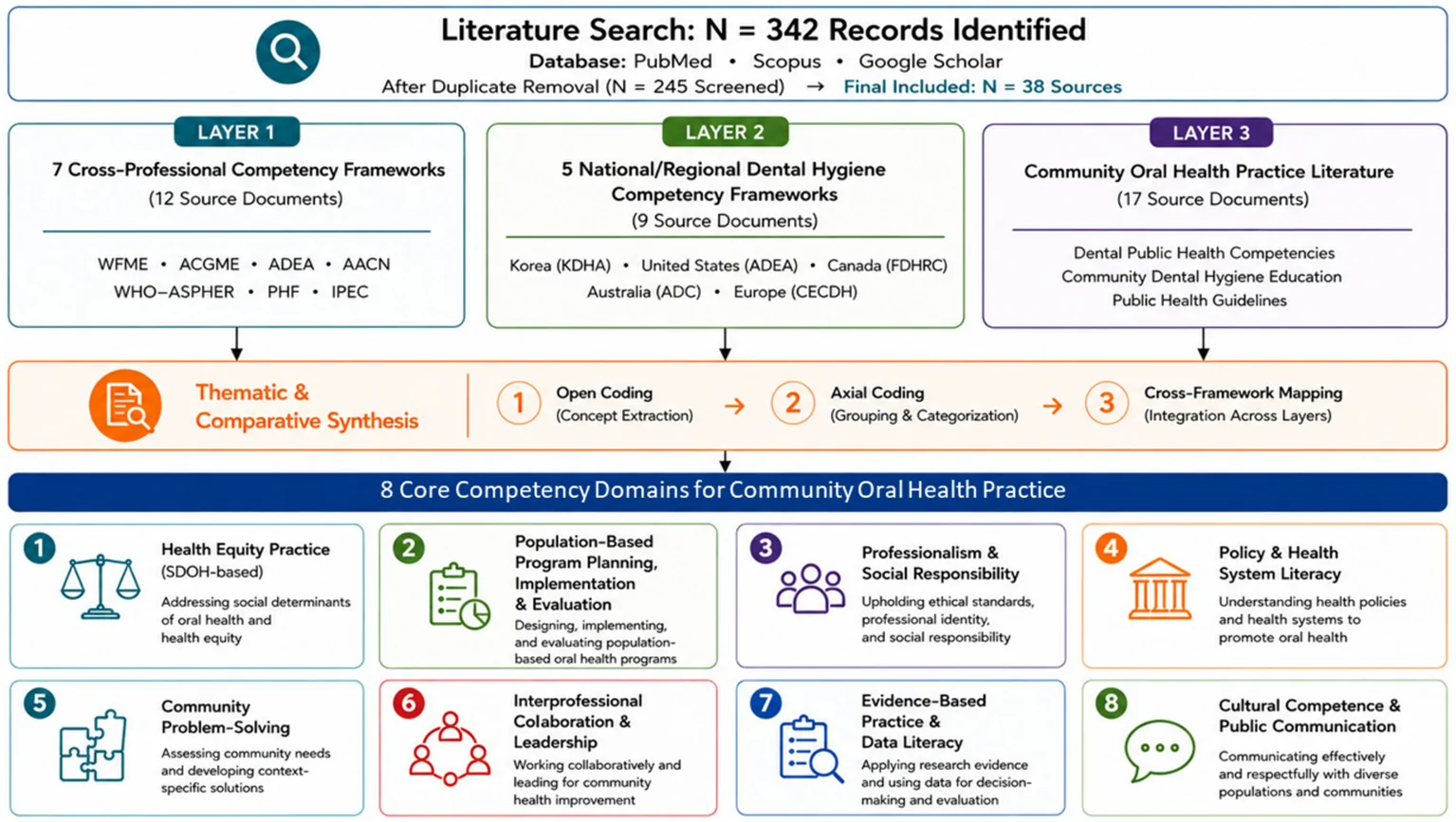
Development of the Community Oral Health Competency Framework through three-layer evidence synthesis. The numbers shown in each analytical layer represent the number of source documents rather than the number of competency frameworks. Layer 1 comprised seven competency frameworks represented by 12 source documents, Layer 2 comprised five national or regional competency frameworks represented by nine source documents, and Layer 3 comprised 17 community oral health practice literature sources.

A total of 342 records were identified through database searching, and 38 sources were included in the final synthesis after duplicate removal and study selection, as described in the Methods section (Figure 1). The included sources were synthesized across three analytical layers: (1) seven internationally recognized competency frameworks for healthcare professionals, including the World Federation for Medical Education (WFME), Accreditation Council for Graduate Medical Education (ACGME), American Dental Education Association (ADEA), American Association of Colleges of Nursing (AACN), WHO–ASPHER Competency Framework for the Public Health Workforce (WHO–ASPHER), Public Health Foundation (PHF), and Interprofessional Education Collaborative (IPEC), analyzed across 12 source documents (*n* = 12); (2) five national or regional competency frameworks for dental hygienists from the Korean Dental Hygienists Association (KDHA), American Dental Education Association (ADEA), Federation of Dental Hygiene Regulators of Canada (FDHRC), Australian Dental Council (ADC), and the Common European Curriculum for Dental Hygiene (CECDH), represented by nine source documents (*n* = 9); and (3) community oral health practice literature, including dental public health competencies, community dental hygiene education, and public health guidelines (*n* = 17). The synthesis process and three-layer evidence structure are illustrated in Figure 2. Cross-layer evidence mapping for all eight competency domains is summarized in Table 1, and the integrated competency framework is presented in Table 2. Detailed evidence mapping for each analytical layer is provided in Supplementary Tables S2–S4.

**Table 1:** Cross-layer evidence mapping for the eight core competency domains.

Competency domain	Layer 1: cross-professional frameworks	Layer 2: national DH frameworks	Layer 3: community practice literature
1. Health Equity Practice (SDOH-based)	WHO-ASPHER, PHF	FDHRC, CECDH, ADC	WHO Social determinants of health, DPH literature
2. Population-Based Program Planning, Implementation & Evaluation	PHF, WHO-ASPHER	ADEA, FDHRC, ADC	ADEA, DPH competencies
3. Professionalism & Social Responsibility	WFME, IPEC, PHF	KDHA, ADEA, FDHRC, ADC, CECDH	Public health ethics literature
4. Policy & Health System Literacy	PHF, WHO-ASPHER	CECDH, FDHRC	PHF, WHO
5. Community Problem-Solving	PHF, IPEC	ADEA, FDHRC, ADC	Community DH education
6. Interprofessional Collaboration & Leadership	IPEC, AACN, PHF	ADEA, FDHRC, ADC, CECDH	IPEC, integrated care literature
7. Evidence-Based Practice & Data Literacy	ACGME, PHF	ADEA, CECDH	Evidence-based practice literature, DPH competencies
8. Cultural Competence & Public Communication	IPEC, WHO-ASPHER	FDHRC, CECDH, ADC	Health promotion frameworks

This table summarizes cross-layer evidence mapping for the eight proposed competency domains. Framework acronyms indicate supporting evidence identified within each analytical layer and do not distinguish whether competency concepts were presented as explicit domains or embedded sub-competencies. Detailed coding of explicit, embedded, and not-identified competency concepts is provided in Supplementary Tables S2–S4.

WFME, World Federation for Medical Education; ACGME, Accreditation Council for Graduate Medical Education; ADEA, American Dental Education Association; AACN, American Association of Colleges of Nursing; WHO-ASPHER, World Health Organization–Association of Schools of Public Health in the European Region; PHF, Public Health Foundation; IPEC, Interprofessional Education Collaborative; KDHA, Korean Dental Hygienists Association; FDHRC, Federation of Dental Hygiene Regulators of Canada; CECDH, Common European Curriculum for Dental Hygiene; ADC, Australian Dental Council. DH, dental hygiene; DPH, dental public health; EBP, evidence-based practice; SDOH, social determinants of health.

**Table 2:** Core competency framework for community oral health practice.

Competency domain	Operational definition	Educational components	Practice implications	Supporting evidence
1. Health Equity Practice (Social determinants of health-based)	Ability to recognize oral health inequities and implement equity-oriented strategies	Mechanisms of oral health disparities; vulnerable population identification; equity-focused intervention design	Reducing oral health disparities through targeted community interventions	WHO-ASPHER; PHF; FDHRC; WHO social determinants of health literature
2. Population-Based Program Planning, Implementation & Evaluation	Ability to assess community needs, design, implement, and evaluate preventive programs	Community needs assessment; program design; implementation and evaluation strategies; quality improvement	Planning and managing community oral health promotion programs	PHF; ADEA; FDHRC
3. Professionalism & Social Responsibility	Ability to apply ethical reasoning and fulfill professional responsibilities in public health contexts	Public health ethics; ethical decision-making; responsibility toward public good and equity	Ethical and accountable practice in community oral health settings	WFME; IPEC; PHF; public health ethics literature
4. Policy & Health System Literacy	Ability to understand health systems and policies and apply them in oral health practice	Healthcare system structures; oral health laws and regulations; policy analysis	Participation in policy implementation and oral health advocacy	PHF; WHO-ASPHER; CECDH; FDHRC
5. Community Problem-Solving	Ability to analyze community oral health issues and develop context-appropriate solutions	Community assessment tools; resource linkage; participatory planning	Developing locally tailored solutions for community oral health issues	PHF; ADEA; FDHRC; community DH literature
6. Interprofessional Collaboration & Leadership	Ability to collaborate across disciplines and lead oral health initiatives within teams	Roles of healthcare professionals; team-based decision-making; leadership skills	Coordinating oral health services within interdisciplinary teams	IPEC; AACN; FDHRC; ADC
7. Evidence-Based Practice & Data Literacy	Ability to appraise evidence and utilize health data and digital tools in practice	Evidence appraisal (EBP); health data analysis; digital health utilization	Implementing data-driven and evidence-based oral health interventions	ACGME; PHF; ADEA; CECDH
8. Cultural Competence & Public Communication	Ability to communicate effectively with diverse populations and deliver culturally appropriate education	Cultural responsiveness; population communication strategies; health education material development	Delivering inclusive oral health education and community engagement	IPEC; WHO-ASPHER; FDHRC; CECDH; ADC

Supporting evidence represents selected key sources for each competency domain and is not intended to provide an exhaustive list of all sources contributing to the domain. Comprehensive cross-layer evidence mapping is presented in Table 1 and Supplementary Tables S2–S4.Abbreviations: AACN, American Association of Colleges of Nursing; ACGME, Accreditation Council for Graduate Medical Education; ADC, Australian Dental Council; ADEA, American Dental Education Association; CECDH, Common European Curriculum for Dental Hygiene; EBP, evidence-based practice; FDHRC, Federation of Dental Hygiene Regulators of Canada; IPEC, Interprofessional Education Collaborative; PHF, Public Health Foundation; WFME, World Federation for Medical Education; WHO-ASPHER, World Health Organization–Association of Schools of Public Health in the European Region.

### 3.2 Competency domains identified across healthcare professional frameworks

This study compared seven internationally recognized competency frameworks spanning medicine, dentistry, nursing, public health, and interprofessional education: WFME, ACGME, ADEA, AACN, WHO-ASPHER, PHF, and IPEC. The paradigm shift from traditional clinical to emerging population health orientations across these frameworks, along with domain-level mapping by framework, is summarized in Supplementary Table S2.

#### 3.2.1 Cross-cutting competency domains

Across the seven frameworks, nine competency domains were identified, including five cross-cutting domains and four framework-specific domains. Of these, five domains were consistently present across all or nearly all frameworks and were classified as cross-cutting: (1) professionalism and ethics, (2) communication and interpersonal skills, (3) critical thinking and evidence-informed practice, (4) health promotion and population health orientation, and (5) interprofessional collaboration. Four domains were identified in select frameworks and are discussed in Section 3.2.2 as framework-specific emphases: systems-based practice, leadership, cultural competence, and patient-centered care.

#### 3.2.2 Framework-specific emphases

Framework emphases differed according to disciplinary orientation and intended educational purpose. Four framework-specific domains were identified: systems-based practice, leadership, cultural competence, and patient-centered care. Clinically oriented frameworks (e.g., WFME, ACGME, ADEA, AACN) placed greater emphasis on patient-centered care and clinical decision-making. In contrast, public health and collaboration-oriented frameworks (e.g., WHO-ASPHER, PHF, IPEC) emphasized systems thinking, policy-related competencies, community engagement, and collaborative practice. Cultural competence and health equity were explicitly stated as independent domains in AACN and WHO-ASPHER, and embedded as sub-competencies within professionalism or population health domains in WFME, ACGME, ADEA, PHF, and IPEC (Supplementary Table S2, Part B).

#### 3.2.3 Structural characteristics relevant to education design

Several frameworks presented explicit educational structures or implementation features. ADEA described curriculum integration strategies such as spiral learning, while AACN presented staged competency progression across learner levels. WHO-ASPHER and PHF specified systems-level competencies including policy development, resource management, and community partnership, and IPEC structured competencies around four domains: shared values and ethics, roles and responsibilities, interprofessional communication, and teamwork.

### 3.3 Core competencies for dental hygienists across countries

Layer 2 comprised nine source documents: one from Korea, three from the United States, two from Canada, one from Australia, and two from Europe. When multiple source documents were available for a single national competency framework, competency concepts were extracted from each document and synthesized at the framework level. Conceptually overlapping competency statements within the same framework were merged and counted once during the synthesis process to minimize duplication while preserving the breadth of competency concepts. Five national or regional dental hygiene competency frameworks were selected for comparative analysis in accordance with the criteria described in Section 2.2.3: Korea (KDHA), the United States (ADEA), Canada (FDHRC), Australia (ADC), and Europe (CECDH). The differences in competency emphasis across national frameworks and the corresponding domain-level mapping are summarized in Supplementary Table S3.

#### 3.3.1 Common competency domains

Across the five frameworks, six domains were consistently identified as common competency areas: (1) communication, (2) interprofessional collaboration, (3) health promotion and community engagement, (4) oral health education, (5) clinical care, and (6) professionalism. These domains were either explicitly stated or embedded across all five national frameworks (Supplementary Table S3, Part B). Three additional domains—leadership, advocacy, and research literacy—were present in select frameworks and varied considerably across countries, as detailed in Section 3.3.2.

#### 3.3.2 Differences in structure and emphasis

Differences were observed in the number of domains and sub-competencies and in the explicit articulation of community- and policy-oriented competencies. Korea’s framework (KDHA) emphasized oral health education and clinical dental hygiene procedures, with comparatively fewer explicitly stated competencies related to policy engagement, leadership, or advocacy. The United States framework included broader domains such as critical thinking, research, advocacy, and practice management. Canada explicitly addressed social justice and cultural competence, including Indigenous health equity, and articulated advocacy as a core domain. Europe (CECDH) included competency areas related to health advocacy, digital ethics, and policy literacy. Australia specified practice expectations reflecting ethical and social sensitivity, such as cultural safety and trauma-informed care. Collectively, the shift from earlier clinic-centered roles toward community coordination, policy participation, and explicit cultural competence reflects a shared professional reorientation documented in Supplementary Table S3, Part A.

#### 3.3.3 Competencies relevant to community oral health practice

When mapped against community oral health practice needs—including equity-oriented prevention, community engagement, and systems/policy awareness—communication, interprofessional collaboration, health promotion and community engagement, and professionalism were consistently positioned as central domains across all five frameworks. In contrast, competencies related to policy engagement, leadership, systems thinking, and digital health were variably described, embedded within other domains, or less explicitly operationalized across countries. This variation reflects the uneven development of community-oriented competency structures internationally and highlights the need for an integrated framework that consolidates these emerging expectations, as proposed in Section 3.5.

### 3.4 Competencies in public oral health and community-based practice literature

To identify competencies directly linked to community oral health practice, key documents and literature in dental public health and community-based oral health education were analyzed. Practice domains and competency component mapping identified across this literature are summarized in Supplementary Table S4.

#### 3.4.1 Common domains

Across sources, five domains were consistently identified across all three literature categories—dental public health specialist literature, community dental hygiene education literature, and public health practice guidelines: (1) oral health program planning and management, (2) understanding healthcare systems and policy, (3) application of oral health policy and law, (4) ethical decision-making in public practice, and (5) public communication for community engagement and partnership (Supplementary Table S4, Part B). An additional five domains—surveillance and data use, interdisciplinary collaboration, evidence-based practice, community research, and SDOH integration—were identified in the majority of sources and represent emerging practice priorities within community-oriented oral health service delivery (Supplementary Table S4, Part A).

#### 3.4.2 Differences by target workforce and scope

Differences were observed between dental public health expert–oriented sources and dental hygienist–oriented sources. Expert-focused sources more frequently specified macro-level competencies, including policy analysis and advocacy, surveillance and data-based monitoring, research design and conduct, and leadership for systems-level interventions. In contrast, dental hygienist–oriented sources emphasized implementation-focused competencies, such as community needs assessment, program delivery, policy application, and community-based education for vulnerable populations. Research-related competencies were more commonly framed as evidence use and practice-based application rather than independent research production. Notably, health equity practice, community partnership, and interprofessional collaboration were consistently identified across all three source categories, underscoring their centrality to community oral health practice regardless of workforce level (Supplementary Table S4, Part B).

### 3.5 Integrated core competencies for community oral health practice

Based on the synthesis of evidence across three analytical layers—as illustrated in Figure 2—an integrated competency framework for community oral health practice was derived. Competency elements that were identified as relevant to equity-oriented and community-based practice across multiple analytical layers were prioritized and consolidated. Cross-layer evidence mapping for each domain is presented in Table 1; notably, all eight domains were supported by convergent evidence across at least two of the three analytical layers, and Professionalism & Social Responsibility was the only domain identified across all five national dental hygiene frameworks (Table 1, Layer 2). The complete framework including operational definitions, educational components, practice implications, and supporting evidence is described in Table 2 (see Supplementary Tables S2–S4 for detailed layer-specific mapping).

#### 3.5.1 Proposed competency domains

Competency concepts identified across the three analytical layers were iteratively compared and grouped according to conceptual similarity. For example, communication-related concepts, including communication, public communication, cultural competence, health communication, and health literacy, were reconceptualized as the domain *“Cultural Competence & Public Communication.”* Likewise, concepts related to health promotion, population health, program planning, implementation, and evaluation, which were consistently identified across international competency frameworks, national dental hygiene frameworks, and community practice literature, were integrated into *“Population-Based Program Planning, Implementation & Evaluation.”* Community assessment, community diagnosis, resource linkage, and participatory planning concepts were synthesized into *“Community Problem-Solving.”* In addition, concepts related to health policy, healthcare systems, and public health policy were consolidated into *“Policy & Health System Literacy,”* whereas evidence-based practice, research utilization, digital health, and health information literacy were synthesized into *“Evidence-Based Practice & Data Literacy.”* Through this iterative synthesis process, the identified competency concepts were consolidated into eight final competency domains (Table 1).

The resulting framework comprises eight competency domains:

1. *Health Equity Practice (Social determinants of health-based):* ability to recognize oral health inequities and implement equity-oriented strategies based on social determinants of health.
2. *Population-Based Program Planning, Implementation & Evaluation:* ability to assess community needs, design, implement, and evaluate preventive programs at the population level.
3. *Professionalism & Social Responsibility:* ability to apply ethical reasoning and fulfill professional responsibilities in public health contexts, with commitment toward the public good and equity. This domain was identified across all five national dental hygiene frameworks.
4. *Policy & Health System Literacy:* ability to understand health systems and policies and apply them within community oral health practice, including healthcare system structures, oral health laws, and policy analysis.
5. *Community Problem-Solving:* ability to analyze community oral health issues and develop context-appropriate, locally tailored solutions using community assessment tools, resource linkage, and participatory planning.
6. *Interprofessional Collaboration & Leadership:* ability to collaborate across disciplines and lead oral health initiatives within multidisciplinary teams, encompassing shared decision-making and leadership skills.
7. *Evidence-Based Practice & Data Literacy:* ability to appraise evidence and utilize health data and digital tools to implement data-driven, evidence-based oral health interventions.
8. *Cultural Competence & Public Communication:* ability to communicate effectively with diverse populations and deliver culturally responsive oral health education and community engagement.

#### 3.5.2 Key structural features of the framework

The proposed framework is distinguished from existing dental hygiene competency models in several important respects. First, it integrates competency evidence derived from cross-professional competency frameworks, national dental hygiene frameworks, and community oral health practice literature, providing a comprehensive competency structure rather than relying on a single disciplinary perspective. Second, it explicitly positions health equity practice based on the social determinants of health as a foundational competency and incorporates population-based program planning, implementation, and evaluation as core professional functions. Third, the framework extends beyond traditional clinical competencies by incorporating Policy & Health System Literacy, Evidence-Based Practice & Data Literacy, Cultural Competence & Public Communication, and Community Problem-Solving. These domains are operationalized through educational components and practice implications that emphasize ethical decision-making, community assessment, program planning and evaluation, policy engagement, data-driven decision-making, interprofessional collaboration, and culturally responsive community engagement. Collectively, these features reflect an expanded professional role for dental hygienists within prevention-focused community oral health practice, extending beyond chairside clinical prevention toward broader public health and population-based practice responsibilities (Table 2).

## 4 Discussion

This study developed a community-oral-health-focused competency framework through a three-layer synthesis of international competency frameworks, national and regional dental hygiene frameworks, and community oral health practice literature. Integrating these complementary sources extends existing competency literature by specifying domains relevant to population-based and community-oriented dental hygiene practice.

The framework aligns with the WHO Global Oral Health Action Plan (GOHAP) 2023–2030, particularly Strategic Objective 2 on health promotion and disease prevention and Strategic Objective 3 on workforce development (16, 21). GOHAP emphasizes social determinants, population-level risk factors, and competency-based workforce models to meet oral health needs at scale (16). Within this policy context, Health Equity Practice (SDOH-based) and Population-Based Program Planning, Implementation & Evaluation reflect Strategic Objective 2, whereas Policy & Health System Literacy and Cultural Competence & Public Communication correspond to workforce priorities under Strategic Objective 3 (4, 22).

The first layer, cross-professional competency frameworks from medicine, nursing, dentistry, and public health, demonstrated a consistent shift from individual clinical care toward prevention-focused, population-based practice. Comparative analyses across health professions report substantial overlap in core competencies and convergence around collaborative values, professional roles, interprofessional communication, and teamwork (23, 24). International interprofessional frameworks such as the WHO Framework for Action on Interprofessional Education and Collaborative Practice and the IPEC Core Competencies similarly highlight collaboration, health equity, systems thinking, and social accountability as central expectations for health professionals (25, 26). In contrast, dental hygiene education has historically emphasized procedure-oriented training with limited measurable competencies in prevention and population health. These observations are also consistent with recent international efforts to redefine the public health role of dental hygienists. Boyd characterized undergraduate oral health education as heavily focused on technical treatment, underscoring the need for curricular reform (27, 28). The eight domains identified in this review respond to that gap by positioning dental hygienists as contributors to prevention-focused community oral health practice while preserving their clinical preventive role, consistent with global oral health workforce frameworks and the direction advocated by the International Federation of Dental Hygienists (9, 15).

The second layer, national dental hygiene competency frameworks from the United States, Canada, Australia, Korea, and Europe, indicates that this professional expansion is underway, though unevenly across regulatory contexts. Beyond Japan, no additional Asian competency frameworks meeting the predefined eligibility criteria were identified.

The Japanese framework was reviewed but did not meet the predefined eligibility criteria because it primarily emphasized educational guidance rather than a standalone competency framework with explicit competency domains and behavioral indicators. Therefore, the relatively limited contribution of the KDHA framework should not be interpreted solely as a characteristic of the Korean framework itself; rather, it may also reflect the current lack of formally published competency frameworks across Asian dental hygiene systems. Competencies related to communication, professionalism, health promotion, and evidence-based practice were generally present, whereas policy literacy, leadership, and cultural responsiveness were articulated more variably—explicit in some frameworks, including the Common European Curriculum for Dental Hygiene (CECDH), and embedded indirectly in others. Bae et al. likewise reported that community and health promotion domains were included but remained less developed than clinical competencies in several international frameworks, indicating an evolving rather than fully consolidated model of community oral health practice (9, 10). The integrated framework proposed here consolidates these emerging expectations into a coherent structure that may support competency development in comparable educational and professional contexts.

The third layer, community oral health practice literature, clarified how these competencies operate in service delivery. Public oral health literature distinguishes macro-level functions such as surveillance and policy development, typically assigned to dental public health specialists, from implementation-level functions such as program delivery and preventive interventions that are more characteristic of dental hygienists (4). The present framework spans these levels: Population-Based Program Planning, Implementation & Evaluation and Cultural Competence & Public Communication support structured community-based service delivery, whereas Community Problem-Solving and Policy & Health System Literacy extend toward broader public health engagement and coordination. This dual capacity is consistent with global oral health competency matrices that integrate direct service provision with public health responsibilities within a single professional framework (15). Previous studies have suggested that dental hygienists working in non-clinical community settings require competencies in advocacy, case management, and cultural responsiveness, underscoring the importance of high-quality training and clearly defined professional roles when providing services to vulnerable and culturally diverse populations (5, 29, 30).

Synthesizing evidence across the three analytical layers indicates that dental hygiene practice is expanding from traditional chairside prevention toward prevention-focused community oral health practice. The framework development process followed established competency synthesis approaches in which recurring constructs across literature, national standards, and practice settings are organized into higher-order domains (31, 32). This approach aligns with prior studies that have derived stable competency structures through cross-mapping and convergence analyses across health professions (23, 33). Domains were retained only when supported by at least two of the three analytical layers (≥2 of 3), thereby ensuring that the resulting framework reflected convergent rather than isolated evidence.

The framework has implications for education, practice, and policy. In education, it supports a shift from procedure-centered training toward prevention-focused community oral health practice and expanded public health responsibilities for dental hygienists (8, 34). This shift requires stronger competency-based education structures and active learning formats such as problem-based learning, community-based practicum, and interprofessional simulation (30, 35, 36). Population-Based Program Planning, Implementation & Evaluation aligns with community-based learning in which students assess needs and design interventions, and the Evidence-Based Practice & Data Literacy domain supports incorporating data and digital tools into coursework (37). Evidence suggests that community-engaged and competency-based learning improves students’ perceived preparedness for public health roles in dental hygiene education (38). In practice and policy, the framework responds to health systems in which community-integrated and long-term care services are expanding, particularly in aging societies (39, 40). Dental hygienists are increasingly expected to deliver prevention-focused services beyond traditional clinical settings for socially vulnerable and older populations (41, 42). The domains of Health Equity Practice (SDOH-based), Community Problem-Solving, and Cultural Competence & Public Communication provide a basis for addressing oral health disparities at the community level (16, 43), while Policy & Health System Literacy enables participation in planning and coordination within integrated care systems, supporting global policy objectives such as GOHAP 2023–2030 (22, 44). In this way, the framework can serve both as an educational reference and as a practical guide for clarifying the professional role of dental hygienists within community oral health practice and for strengthening prevention-focused services (45). Its application will nonetheless require contextual adaptation and further validation in specific sociocultural and healthcare settings.

This study has several limitations. As a scoping review, it was designed to map and synthesize evidence rather than evaluate methodological quality or intervention effects (19). Accordingly, the proposed framework should be interpreted as a conceptually integrated structure that requires further empirical validation through stakeholder consensus and field-based evaluation. The literature search was conducted between March and April 2025, and recently published competency frameworks or revised national standards after the search period may not have been captured. Restriction to English- and Korean-language sources and the requirement for full-text accessibility may have excluded relevant documents from other linguistic and healthcare contexts. The purposive selection of five national frameworks and the exclusion of Japan and the United Kingdom may have introduced selection bias and limited the geographic representativeness of Layer 2 findings. In addition, the Common European Curriculum for Dental Hygiene (CECDH) was used as a regional framework despite substantial variation in regulation, educational standards, professional recognition, and scope of practice across European countries; therefore, findings derived from the CECDH framework should not be interpreted as representing all European contexts. The three analytical layers differed in document type, scope, and evidential weight, and the ≥2 of 3 convergence criterion represented a pragmatic analytic decision that may have obscured structural differences among evidence sources. Google Scholar also has inherent limitations, including dynamic indexing, limited search reproducibility, and the absence of systematic export functions. Furthermore, competency-domain categorization involved interpretive qualitative synthesis and iterative consensus-based refinement rather than discrete binary screening decisions. Consequently, quantitative inter-rater agreement statistics were not considered methodologically appropriate for this stage, although the absence of quantitative agreement measures remains a limitation of the study. Finally, because most included sources originated from high-income, predominantly Anglophone countries, the proposed framework may require further validation and contextual adaptation before application in different sociocultural, educational, and healthcare-system environments.

Future research should focus on validating and refining the proposed competency framework through expert consensus methods, such as Delphi studies, and empirical evaluation in educational and practice settings. Additional studies across diverse sociocultural, educational, and healthcare-system contexts, particularly in low- and middle-income countries and underrepresented regions, are needed to examine the framework’s transferability and contextual applicability. Furthermore, longitudinal research evaluating competency-based curriculum implementation and workforce outcomes would strengthen the evidence supporting its practical use in community oral health practice.

Despite these limitations, by synthesizing evidence across cross-professional competency frameworks, national and regional dental hygiene competency frameworks, and community oral health practice literature, this review provides a community-oral-health-specific integrated competency framework for dental hygienists and represents a novel application of a three-layer synthesis to this workforce-specific domain.

## 5 Conclusion

This scoping review identified core competencies for community oral health practice among dental hygienists by synthesizing evidence from cross-professional competency frameworks, national dental hygiene competency frameworks, and community oral health practice literature into an integrated eight-domain framework. The resulting domains—Health Equity Practice (SDOH-based), Population-Based Program Planning, Implementation & Evaluation, Professionalism & Social Responsibility, Policy & Health System Literacy, Community Problem-Solving, Interprofessional Collaboration & Leadership, Evidence-Based Practice & Data Literacy, and Cultural Competence & Public Communication—provide a structured competency framework that reflects the expanding role of dental hygienists in prevention-focused community oral health practice. Collectively, these findings demonstrate that contemporary community oral health practice requires competencies extending beyond traditional clinical prevention to encompass population health, health equity, policy engagement, and community-based practice. Although the framework is conceptual and requires empirical validation through expert consensus processes, such as Delphi studies, and field-based testing, it may provide a practical foundation for curriculum reform, accreditation standards, and workforce policy development in educational and professional settings similar to those represented in the included sources. Future research should further validate and refine the framework across diverse sociocultural, educational, and healthcare-system contexts to support its broader implementation.

## Data Availability

All data generated or analyzed during this study are included in this article and its Supplementary Material.

## References

[1] Petersen PE Kwan S. Equity, social determinants and public health programmes—the case of oral health. Community Dent Oral Epidemiol. (2011) 39:481–7. doi: 10.1111/j.1600-0528.2011.00623.x, 21623864

[2] Gallagher JE Mattos Savage GC Crummey SC Sabbah W Makino Y Varenne B. Health workforce for oral health inequity: opportunity for action. PLoS One. (2024) 19:e0292549. doi: 10.1371/journal.pone.0292549, 38870162 PMC11175420

[3] Monajem S. Integration of oral health into primary health care: the role of dental hygienists and the WHO stewardship. Int J Dent Hyg. (2006) 4:47–51. doi: 10.1111/j.1601-5037.2006.00159.x, 16451440

[4] Mascarenhas AK Atchison KA. Developing core dental public health competencies for predoctoral dental and dental hygiene students. J Public Health Dent. (2015) 75:S6–S11. doi: 10.1111/jphd.12129, 26630639

[5] Park SK Kim NH. Exploring core competencies of nonclinical dental hygienists: implications for social dental hygiene curriculum development. J Korean Soc Dent Hyg. (2025) 25:241–53. doi: 10.13065/jksdh.2025.25.3.7

[6] Malekmohammadi M Ghasemi H Khoshnevisan MH Hosseini F. Competencies for dental public health specialists: a thematic analysis. Eur J Dent Educ. (2023) 27:928–40. doi: 10.1111/eje.12883, 36519508

[7] Annamma LM Varma SR Abuttayem H Prasad P Azim SA Odeh R . Current challenges in dental education: a scoping review. BMC Med Educ. (2024) 24:1523. doi: 10.1186/s12909-024-06545-1, 39716191 PMC11667986

[8] Yoo SH Bae SM Shin BM Shin SJ. The effect of project-based learning modules on a community dental hygiene practicum in South Korea. J Dent Educ. (2020) 84:418–28. doi: 10.1002/jdd.12047, 32030762

[9] Bae SM Chung WG Jang JH Mun SJ Shin BM Shin SJ. Competencies for entry into the profession of dental hygiene. J Dent Hyg Sci. (2017) 17:193–201. doi: 10.17135/jdhs.2017.17.3.193

[10] Jongbloed-Zoet C Nyblom Y Bol E Field JC. A common European curriculum for dental hygiene. Eur J Dent Educ. (2020) 24:611–5. doi: 10.1111/eje.12501, 32949438 PMC7693029

[11] Luciak-Donsberger C Eaton KA. Dental hygienists in Europe: trends towards harmonization of education and practice since 2003. Int J Dent Hyg. (2009) 7:273–84. doi: 10.1111/j.1601-5037.2009.00402.x, 19832915

[12] Johnson PM. International profiles of dental hygiene 1987 to 2006: a 21-nation comparative study. Int Dent J. (2009) 59:63–77. doi: 10.1922/IDJ_2076Johnson15, 19489285

[13] Jiang X Ding Z Wang F Wang Z Wang W Xing Y . Construction of a competency framework of dental hygienists: a Delphi study. Nurse Educ Pract. (2023) 70:103692. doi: 10.1016/j.nepr.2023.103692, 37379696

[14] Park EJ. Accreditation standards in dental hygiene education: global comparison and implications. J Dent Hyg Sci. (2025) 25:227–36. doi: 10.17135/jdhs.2025.25.3.227

[15] Benzian H Greenspan JS Barrow J Hutter JW Loomer PM Stauf N . A competency matrix for global oral health. J Dent Educ. (2015) 79:353–61. doi: 10.1002/j.0022-0337.2015.79.4.tb05891.x, 25838005

[16] Naresh A McGrath R England R Wanyonyi-Kay K Kumar V Ram A . Redefining the oral health workforce: advancing equity and improving global oral health outcomes through inclusive, strategic reform. Front Dent Med. (2025) 6:1670715. doi: 10.3389/fdmed.2025.1670715, 41199792 PMC12586154

[17] Peters MD Godfrey CM Khalil H McInerney P Parker D Soares CB. Guidance for conducting systematic scoping reviews. JBI Evid Implement. (2015) 13:141–6. doi: 10.1097/XEB.0000000000000050, 26134548

[18] Khalil H Peters MD Tricco AC Pollock D Alexander L McInerney P . Conducting high quality scoping reviews: challenges and solutions. J Clin Epidemiol. (2021) 130:156–60. doi: 10.1016/j.jclinepi.2020.10.009, 33122034

[19] Arksey H O'Malley L. Scoping studies: towards a methodological framework. Int J Soc Res Methodol. (2005) 8:19–32. doi: 10.1080/1364557032000119616

[20] Peters MD Marnie C Tricco AC Pollock D Munn Z Alexander L . Updated methodological guidance for the conduct of scoping reviews. JBI Evid Synth. (2020) 18:2119–26. doi: 10.11124/JBIES-20-00167, 33038124

[21] Mehta A Mathur M Watt R Guarnizo-Herreño CC Mutave RJ Goes PS . Future-proofing the oral health workforce: evidence from four low- and middle-income countries on training needs in dental public health. Cureus. (2025) 17:e96276. doi: 10.7759/cureus.96276, 41362563 PMC12681956

[22] Eaton K Yusuf H Vassallo P. The WHO global oral health action plan 2023–2030. Community Dent Health. (2023) 40:68–9. doi: 10.1922/CDH_Jun23Editorial02, 37265395

[23] Spielman AI Fulmer T Eisenberg ES Alfano MC. Dentistry, nursing, and medicine: a comparison of core competencies. J Dent Educ. (2005) 69:1257–71. doi: 10.1002/j.0022-0337.2005.69.11.tb04025.x, 16275689

[24] Thistlethwaite JE Forman D Matthews LR Rogers GD Steketee C Yassine T. Competencies and frameworks in interprofessional education: a comparative analysis. Acad Med. (2014) 89:869–75. doi: 10.1097/ACM.0000000000000249, 24871237

[25] Gilbert JHV Yan J Hoffman SJ. A WHO report: framework for action on interprofessional education and collaborative practice. J Allied Health. (2010) 39:196–7. 21174039

[26] Interprofessional Education Collaborative Expert Panel. Core Competencies for Interprofessional Collaborative Practice: Report of an Expert Panel. Washington, DC: Interprofessional Education Collaborative Expert Panel (2011).

[27] Boyd LD. Educating dental hygienists to meet future health care needs and roles of the profession. J Dent Educ. (2016) 80:1031–2. doi: 10.1002/jdd.12228, 27587569

[28] Fried JL Maxey HL Battani K Gurenlian JR Byrd TO Brunick A. Preparing the future dental hygiene workforce: knowledge, skills, and reform. J Dent Educ. (2017) 81:eS45–52. doi: 10.21815/JDE.017.032, 28864803

[29] Beatty CF. Community Oral Health Practice for the Dental Hygienist-E-Book. 5th ed. St. Louis, MO: Elsevier Health Sciences (2021).

[30] Yoon M Compton S. Building professional competence in dental hygiene students through a community-based practicum. Int J Dent Hyg. (2017) 15:e119–27. doi: 10.1111/idh.12233, 27256858

[31] Kilani MA Rodriguez-Feria P Pavlova M Krasna H Aljohar BA Aragon de Leon E . Research methodologies for creating competency frameworks for the public health workforce: a scoping review. Eur J Pub Health. (2026) 36:i8–i13. doi: 10.1093/eurpub/ckaf237, 41400839

[32] MacKay M Ford C Grant LE Papadopoulos A McWhirter JE. Developing public health competency statements and frameworks: a scoping review and thematic analysis of approaches. BMC Public Health. (2023) 23:2240. doi: 10.1186/s12889-023-17182-6, 37957658 PMC10644570

[33] Batt AM Tavares W Williams B. The development of competency frameworks in healthcare professions: a scoping review. Adv Health Sci Educ. (2020) 25:913–87. doi: 10.1007/s10459-019-09946-w, 31797195

[34] Rowley LJ Stein SM. A baccalaureate education curriculum to prepare dental hygienists for expanded public health practice. J Evid Based Dent Pract. (2016) 16:122–8. doi: 10.1016/j.jebdp.2016.01.024 27237005

[35] Moore TS. Implementation of problem-based learning in a baccalaureate dental hygiene program. J Dent Educ. (2007) 71:1058–69. doi: 10.1002/j.0022-0337.2007.71.8.tb04372.x, 17687088

[36] Henesy MA Henderson RP Gross EL Shah A Kearney RC. Dental and dental hygiene students’ perceptions on intraprofessional education. J Dent Educ. (2025) 89:355–62. doi: 10.1002/jdd.13741, 39390702 PMC11903920

[37] Nazeha N Pavagadhi D Kyaw BM Car J Jimenez G Car LT. A digitally competent health workforce: scoping review of educational frameworks. J Med Internet Res. (2020) 22:e22706. doi: 10.2196/22706, 33151152 PMC7677019

[38] McFarland KK Nayar P Ojha D Chandak A Gupta N Lange B. Impact of community-based dental education on attainment of ADEA competencies: students’ self-ratings. J Dent Educ. (2016) 80:670–6. doi: 10.1002/j.0022-0337.2016.80.6.tb06128.x, 27251348

[39] West K Saunders J Slack-Smith L. Integrating dental professionals into aged care with focus on Australia: a scoping review. Gerodontology. (2025) 42:147–64. doi: 10.1111/ger.12784, 39800348 PMC12106945

[40] Oishi MM Childs CA Gluch JI Marchini L. Delivery and financing of oral health care in long-term services and supports: a scoping review. J Am Dent Assoc. (2021) 152:215–223.e2. doi: 10.1016/j.adaj.2020.12.004, 33632411

[41] Lee SH Bae SM Shin BM Shin SJ. Perceptions of visiting oral health service providers: using the Q method and focusing on suggestions for the role of dental hygienists in a community. Gerodontology. (2025) 42:545–53. doi: 10.1111/ger.12824, 40418182 PMC12606390

[42] Baltutis L Gussy M Morgan M. The role of the dental hygienist in the public health sector; an Australian perspective. Int Dent J. (2000) 50:29–35. doi: 10.1111/j.1875-595X.2000.tb00543.x, 10945177

[43] Glassman P Harrington M Namakian M. Promoting oral health through community engagement. J Calif Dent Assoc. (2014) 42:465–70. doi: 10.1080/19424396.2014.12221388, 25076629

[44] Fisher-Owens SA Barker JC Adams S Chung LH Gansky SA Hyde S . Giving policy some teeth: routes to reducing disparities in oral health. Health Aff. (2008) 27:404–12. doi: 10.1377/hlthaff.27.2.404, 18332496

[45] Ahern J. Advancing oral health equity through medical-dental integration: dental hygienists as catalysts for change in an evolving health care system. J Dent Hyg. (2024) 98:8–12. 38876794

[46] Tricco AC Lillie E Zarin W O’Brien KK Colquhoun H Levac D . PRISMA extension for scoping reviews (PRISMA-ScR): checklist and explanation. Ann Intern Med. (2018) 169:467–73. doi: 10.7326/M18-0850 30178033

