# Supplementary material for: Identifying core competencies for community oral health practice among dental hygienists: a scoping review
Source: Front Public Health. 2026 Sep 9;14:1877748. doi: 10.3389/fpubh.2026.1877748 (PMC13597712; doi:10.3389/fpubh.2026.1877748)
Supplement: Supplementary file 1 [file Data_Sheet_1.docx]

**Supplementary Appendix S1. Database-Specific Search Strategies**

Search strategies were developed for each database using Boolean operators, phrase searching, and database-specific field restrictions, as appropriate.

Search period: March 1, 2025 – April 10, 2025

**Database: PubMed**

Search strategy: (("core competencies"[Title/Abstract] OR "professional competencies"[Title/Abstract] OR competency[Title/Abstract]) AND ("dental hygiene"[Title/Abstract] OR "oral health"[Title/Abstract] OR "community oral health"[Title/Abstract]) AND ("public health"[Title/Abstract] OR "health promotion"[Title/Abstract] OR "health equity"[Title/Abstract] OR advocacy[Title/Abstract] OR policy[Title/Abstract] OR "cultural competence"[Title/Abstract] OR "interprofessional collaboration"[Title/Abstract] OR "digital health"[Title/Abstract]))
Search limits:

• Publication year: 2005–2025

• Language: English

MeSH terms were not used because the review aimed to comprehensively capture competency-related concepts, which are not consistently indexed using Medical Subject Headings (MeSH). Therefore, a free-text search restricted to the Title/Abstract fields was performed.

**Database: Scopus**

Search strategy: TITLE-ABS
 (("core competencies" OR "professional competencies" OR competency) AND ("dental hygiene" OR "oral health" OR "community oral health") AND ("public health" OR "health promotion" OR "health equity"
OR advocacy OR policy OR "cultural competence" OR "interprofessional collaboration" OR "digital health"))

Search limits:

• Publication year: 2005–2025

• Language: English

Scopus searches were conducted using the TITLE-ABS field. Truncation operators were not applied because the selected search terms represented standardized competency terminology, and preliminary searches indicated that adequate retrieval was achieved without wildcard expansion.

**Database: Google Scholar**

Search strategy: ("core competencies" OR "professional competencies") AND ("dental hygiene" OR "oral health" OR "community oral health") AND ("public health" OR "health promotion" OR "health equity" OR advocacy OR policy OR "cultural competence" OR "interprofessional collaboration" OR "digital health")

Search limits:

- Publication year: 2005–2025
- Language: English and Korean

Screening procedure

The first 100 records sorted by relevance were manually screened according to the predefined eligibility criteria. Reference lists of eligible publications were also reviewed to identify additional competency frameworks and policy documents.

**Additional Searches**

Candidate competency frameworks were identified through targeted searches of official websites of professional associations, accreditation organizations, and competency framework developers. Searches focused on competency frameworks relevant to health professions education, public health, oral health, and dental hygiene. Retrieved documents were subsequently assessed according to the predefined eligibility criteria described in Section 2.2.3.

Targeted searches of official websites of professional associations, accreditation organizations, and regulatory bodies were conducted to identify competency frameworks, guidelines, and policy documents that were not indexed in the bibliographic databases.

**Supplementary Table S1. Summary of targeted website searches used to identify candidate competency frameworks**

| **Website** | **Search terms** | **Search date** | **Outcome** |
| --- | --- | --- | --- |
| Korean Dental Hygienists Association (KDHA) | "competency", "competency framework", "dental hygiene" | April 10, 2025 | Relevant documents screened |
| American Dental Education Association (ADEA) | "competency framework", "dental hygiene competencies" | April 10, 2025 | Relevant documents screened |
| Federation of Dental Hygiene Regulators of Canada (FDHRC) | "entry-to-practice competencies", "competency framework" | April 10, 2025 | Relevant documents screened |
| Australian Dental Council (ADC) | "professional competencies", "dental hygiene" | April 10, 2025 | Relevant documents screened |
| Common European Curriculum for Dental Hygiene (CECDH) | "curriculum", "competencies", "dental hygiene" | April 10, 2025 | Relevant documents screened |

Documents retrieved through targeted website searches were screened according to the predefined eligibility criteria described in Section 2.2.5. Frameworks meeting all four predefined eligibility criteria were included in Layer 2 of the synthesis.

**Supplementary Table S2. Layer 1 Analysis of Cross-Professional Competency Frameworks**

**Part A. Evolution of Competency Paradigms**

| **Competency Domain** | **Traditional Clinical Paradigm** | **Emerging Population Health Paradigm** |
| --- | --- | --- |
| Professionalism | Professional responsibility | Social accountability and public role |
| Communication | Provider–patient interaction | Community engagement |
| Evidence-based practice | Clinical decision-making | Population data interpretation |
| Health promotion | Preventive counseling | Population health intervention |
| Interprofessional collaboration | Referral and teamwork | Intersectoral coordination |
| Systems-based practice | Healthcare delivery context | Policy and system understanding |
| Leadership | Clinical leadership | Community and organizational leadership |

* The term "social accountability" appears only as a source-derived concept and was synthesized into the final domain label "Professionalism & Social Responsibility."

**Part B. Framework-Level Coding of Competency Domains**

| **Competency Domain** | **WFME** | **ACGME** | **ADEA** | **AACN** | **WHO-ASPHER** | **PHF** | **IPEC** |
| --- | --- | --- | --- | --- | --- | --- | --- |
| Professionalism & ethics | ✓ | ✓ | ✓ | ✓ | ✓ | ● | ✓ |
| Communication | ✓ | ✓ | ✓ | ✓ | ✓ | ✓ | ✓ |
| Critical thinking / Evidence-based practice BP | ✓ | ✓ | ✓ | ✓ | ● | ✓ | – |
| Population health | ● | – | ● | ✓ | ✓ | ✓ | – |
| Interprofessional collaboration | ● | ● | ● | ✓ | ✓ | ● | ✓ |
| Policy & system understanding | – | – | – | ● | ✓ | ✓ | – |
| Cultural competence / equity | ● | ● | ● | ✓ | ✓ | ● | ✓ |
| Systems-based practice | – | ✓ | – | ● | ✓ | ✓ | – |
| Leadership | – | – | ● | ✓ | ● | ✓ | – |

✓ Explicitly stated domain ● Domain embedded within sub-competencies – Domain not identified

**Supplementary Table S3. Layer 2 Analysis of National Dental Hygiene Competency Frameworks
Part A. Shift in Competency Emphasis Across National Frameworks**

| **Competency Domain** | **Earlier Emphasis** | **Recent Emphasis** |
| --- | --- | --- |
| Communication | Patient instruction | Community communication |
| Collaboration | Dental team cooperation | Interprofessional collaboration |
| Health promotion | Preventive advice | Population-based prevention |
| Ethics & responsibility | Professional conduct | Advocacy and social accountability |
| Leadership | Clinic management | Community coordination |
| Policy understanding | Minimal | Policy participation |
| Cultural competence | Implicit | Explicit competency |

**Part B. Cross-National Competency Mapping**

| **Competency** | **Korea (KDHA)** | **USA (ADEA)** | **Canada (FDHRC)** | **Europe (CECDH)** | **Australia (ADC)** |
| --- | --- | --- | --- | --- | --- |
| Communication | ✓ | ✓ | ✓ | ✓ | ✓ |
| Health promotion | ✓ | ✓ | ✓ | ✓ | ✓ |
| Clinical care | ✓ | ✓ | ✓ | ✓ | ✓ |
| Professionalism | ✓ | ✓ | ✓ | ✓ | ✓ |
| Leadership | – | ✓ | ✓ | ✓ | ✓ |
| Policy engagement | – | ● | ✓ | ✓ | ● |
| Cultural competence | ● | ✓ | ✓ | ✓ | ✓ |
| Advocacy | – | ✓ | ✓ | ✓ | ● |
| Research literacy | – | ✓ | ● | ● | ● |

✓ Explicitly stated ● Embedded – Not identified

**Supplementary Table S4. Layer 3 Analysis of Community Oral Health Practice Literature
Part A. Practice Domains in Community-Oriented Literature**

| **Practice Domain** | **Core Capability** | **Community Health Function** |
| --- | --- | --- |
| Program planning | Needs assessment & evaluation | Community prevention programs |
| Health system literacy | Structural analysis | Access improvement |
| Ethical decision-making | Equity-based judgment | Public accountability |
| Surveillance & data use | Indicator monitoring | Evidence-informed intervention |
| Public communication | Community engagement | Health literacy improvement |
| Interdisciplinary collaboration | Coordinated care | Integrated services |
| Policy application | Advocacy | Prevention policy implementation |
| Evidence-based practice | Scientific evidence use | Effective intervention |
| Community research | Program evaluation | Continuous improvement |
| Social determinants of health integration | Determinant-based approach | Health equity improvement |

**Part B. Competency Component Mapping**

| **Competency Component** | **DPH Specialists Literature** | **Community DH Education** | **Public Health Guidelines** |
| --- | --- | --- | --- |
| Program planning & evaluation | ✓ | ✓ | ✓ |
| Community needs assessment | ✓ | ✓ | ✓ |
| Policy understanding | ✓ | ● | ✓ |
| Policy application | ● | ✓ | ✓ |
| Health equity practice | ✓ | ✓ | ✓ |
| Ethical decision-making | ✓ | ✓ | ● |
| Interprofessional collaboration | ● | ✓ | ✓ |
| Community partnership | ✓ | ✓ | ✓ |
| Education & behavior change | ● | ✓ | ✓ |
| Evidence use | ✓ | ✓ | ● |

✓ Explicitly stated ● Embedded – Not identified
